# Performance of a genetic algorithm for mass spectrometry proteomics

**DOI:** 10.1186/1471-2105-5-180

**Published:** 2004-11-19

**Authors:** Neal O Jeffries

**Affiliations:** 1 Office of the Clinical Director, National Institute of Neurological Disorders and Stroke, Bethesda MD, USA

## Abstract

**Background:**

Recently, mass spectrometry data have been mined using a genetic algorithm to produce discriminatory models that distinguish healthy individuals from those with cancer. This algorithm is the basis for claims of 100% sensitivity and specificity in two related publicly available datasets. To date, no detailed attempts have been made to explore the properties of this genetic algorithm within proteomic applications. Here the algorithm's performance on these datasets is evaluated relative to other methods.

**Results:**

In reproducing the method, some modifications of the algorithm as it is described are necessary to get good performance. After modification, a cross-validation approach to model selection is used. The overall classification accuracy is comparable though not superior to other approaches considered. Also, some aspects of the process rely upon random sampling and thus for a fixed dataset the algorithm can produce many different models. This raises questions about how to choose among competing models. How this choice is made is important for interpreting sensitivity and specificity results as merely choosing the model with lowest test set error rate leads to overestimates of model performance.

**Conclusions:**

The algorithm needs to be modified to reduce variability and care must be taken in how to choose among competing models. Results derived from this algorithm must be accompanied by a full description of model selection procedures to give confidence that the reported accuracy is not overstated.

## Background

When Petricoin et al. [1] published their analysis using serum to distinguish individuals with ovarian cancer from individuals with benign conditions, it suggested great promise in using high throughput mass spectrometry to improve upon existing biomarkers for patient groups that could greatly benefit from accurate and early diagnosis. Results from the analyses using this algorithm have, along with early findings from other groups (e.g. [2,3]), fueled an explosion of interest in using mass spectrometry techniques for quick and accurate diagnosis. Many other investigators have since used classification techniques to achieve impressive results in correctly categorizing unlabeled mass spectrometry samples as either diseased or healthy. Among the more common classification methods used are classification trees [2], boosting [4], stepwise discrimination methods [5], and wavelet discrimination [6], though few have used a genetic algorithm. Baggerly et al. [7] do use a genetic algorithm though its properties are substantially different than that used in the earlier studies and is generally not subject to the conclusions drawn below. Here we evaluate the performance of a genetic algorithm described in the original Lancet paper and subsequent studies [8,9].

### Description of the algorithm

The specifics of the genetic algorithm used here are based on webpages at the NCI-FDA website housing the data [10] and [1,9]. As the descriptions are limited, this attempt to reproduce the algorithm may differ from the implementation supporting the published results.

• As described, the genetic algorithm (GA) seeks to find a collection of markers that separate cases and controls. Here the markers correspond to a biological sample's measurements at a given set of *m*/*z *values. In the GA framework such a collection of markers is called a chromosome. Each chromosome is evaluated by a fitness function in the following way. Suppose a given chromosome is composed of *N *"genes" (i.e. *N m/z *values in this case). Each sample's intensity values at the *N *genes are linearly scaled to lie between 0 and 1; the smallest of the *N *intensities is assigned 0, the largest assigned 1, and intermediate values interpolated in a linear way. The first sample is assigned its own cluster with centroid (i.e. mean values) given by its *N *values. The next sample is compared to the first and if the Euclidean distance between the two samples exceeds .1·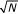 then the second sample is assigned a new cluster with centroid given by its values. If the distance is less than this limit the second sample is assigned to the first cluster and the centroid values recalculated as the mean of both cases. Subsequent cases are handled similarly – if a sample's representation as an *N *dimensional point lies within the .1·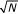 limit of a cluster the case is assigned to the closest cluster and centroid values are recalculated. If the smallest distance to any cluster centroid exceeds the limit then the case is assigned a new cluster. When all the cases are clustered a cluster's type is designated by majority vote – those clusters composed mostly of cancer cases are deemed cancer clusters and those composed mostly of nondiseased are likewise defined. The fitness function then computes the chromosome's fitness as its classification accuracy, i.e. the proportion of cases assigned to a cluster of the appropriate type.

• The selection process starts with 1500 randomly chosen chromosomes, i.e. sets of markers. The documentation indicates that chromosomes with length between 5 and 20 markers are used. In this implementation, different chromosomes may have different lengths – the 1500 are chosen to have a length between 5 and 20 with a uniform probability of 1/16 governing the choice. It is not clear how the original GA treated chromosomes of different length.

Each of the 1500 chromosomes is evaluated by the fitness function as described above. Chromosome pairs are then produced with the likelihood of being selected for a pair related to the fitness function. The available documentation does not make clear how this probability is explicitly related to the fitness. Here the choice was made by ranking the fitness scores and setting parameters *α *and *β *such that

*Prob *[ selecting *k*^*th *^ranked chromosome ] = *α *+ *βk*

where *α *and *β *were chosen such that


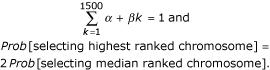


This approach is described in section 2.1 of [11] – ranking is preferred to absolute fitness values as it avoids some potential problems with scaling. An alternative method based on absolute fitness values produced qualitatively similar findings (results not shown).

• A new generation of chromosomes is produced by first creating 750 parent pairs from the set of 1500 chromosomes. A parent pair is created by choosing two of the 1500 chromosomes using the above probabilities. A given chromosome can be chosen to be in more than one pair. For a given pair each chromosome is broken to produce two sub-chromosomes. The location of the break is random with uniform probabilities. The two sets of sub-chromosomes are then crossed-over to produce two new chromosomes. As an example suppose chromosome 1 has genes = (3001, 5500, 7800, 11011, 13059) and chromosome 2 is composed of *m*/*z *locations = (2500, 4200, 909, 15002) and the first chromosome breaks between its second and third elements while the second chromosome breaks after its third. Then the resulting new chromosomes are (3001, 5500, 15002) and (2500, 4200, 909, 7800, 11011, 13059). In this way the 750 pairs of chosen chromosomes produce a next generation of 1500 chromosomes. At this point each gene in each new chromosome may be randomly changed to any other gene in the entire spectrum range with probability .0002 (this corresponds to genetic mutation). In our implementation it is possible to match sub-chromosomes that would merge to be longer than 20 units. In this event the chromosome is truncated to 20. Further, here it is allowed to have chromosomes composed of as few as 2 markers as there seemed no compelling reason to impose a lower limit of 5 *m*/*z *values as described in the documentation. It is unclear if the original GA allowed chromosomes of different lengths to reproduce or instead restricted cross-overs to pairs with the same length.

• After mutation this new generation of 1500 chromosomes is then evaluated by the fitness test and then another round of selection, cross-over, and mutation processes produce the next generation. Typically, the average fitness of generations increases over time. According to the documentation, the process stops 1) after 250 generations, or 2) when a perfectly discriminating chromosome is found. In the first instance, that model that has produced the highest fitness score is chosen.

• Given a chosen model derived from a training set, an unlabeled spectrum (e.g. test set spectrum) is classified by determining that cluster with the centroid that lies closest to the unlabeled case and assigning the label of the cluster. The documentation describes a second, related approach that is to make this assignment only if the nearest centroid is with .1·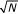; otherwise assign the case as of a third, unknown/new type. In this current work, classification errors for a test set correspond to the first criteria of nearest centroid, without the .1·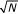 requirement. Empirically, this led to greater classification accuracy.

Some concerns about this algorithm have been raised by others [7]; a few issues will be examined below in more detail.

• Each chromosome is evaluated by a fitness function that measures how well the chromosome classifies the training set. However it is clear that the order in which the cases are considered may make a difference in what cluster a case is assigned as well as the clusters' centroid values. Consequently, different results may arise in attempts to replicate findings.

• The GA algorithm starts with a random selection of 1500 chromosomes and then letting these evolve through a random mating process. As the initial selection and evolutionary process is random it is again the case that different ultimate models may be chosen, depending on the seed of a random number generator.

• In this application, each chromosome partitions the samples of the training set into clusters defined as groups of cases with centroids that are at least .1·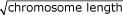 apart from one another. It is not uncommon to find chromosomes that partition perfectly, but rely upon a large number of clusters (e.g. > 30). This suggests overfitting of data. As described, this algorithm does not penalize or otherwise take into account the number of clusters or length of chromosomes.

Largely because of insufficient information, this implementation of the algorithm likely differs from that supporting the published results. However, some elements should be the same. In particular, the evaluation of the fitness function should yield the same results except for the issue of how the order of the cases can change the clusters' attributes. Therefore, we should be able to match or come close to verifying the published results for a given model. However, our results are likely to be different as far as generating best models. This is in part due to the inherent randomness the process employs as well as possible differences in how the fitness function scores generate members of the next generation.

### Datasets

Two publicly available datasets were used to evaluate the algorithm; information regarding them is available from an NCI-FDA website [10]. Both datasets consist of ovarian cancer patients and healthy controls. The first dataset, hereafter referred to as DS1, contains "low resolution" mass spectrometry data from a Ciphergen instrument and is identified on the NCI-FDA website as the 8-7-02 data. The data consist of 162 ovarian cancer samples and 91 control samples. The second dataset, DS2, contains "high resolution" data from a hybrid quadrupole time-of-flight spectrometer. Description and analysis of these data are available in [12]. The dataset contains spectra from 121 cancer samples and 95 controls.

For both datasets GA-produced models are presented on the NCI-FDA website that were developed from a training set and perfectly discriminate a test set. There is no designation as to which individuals were used for training and which for testing.

## Results

**Figure 1:**
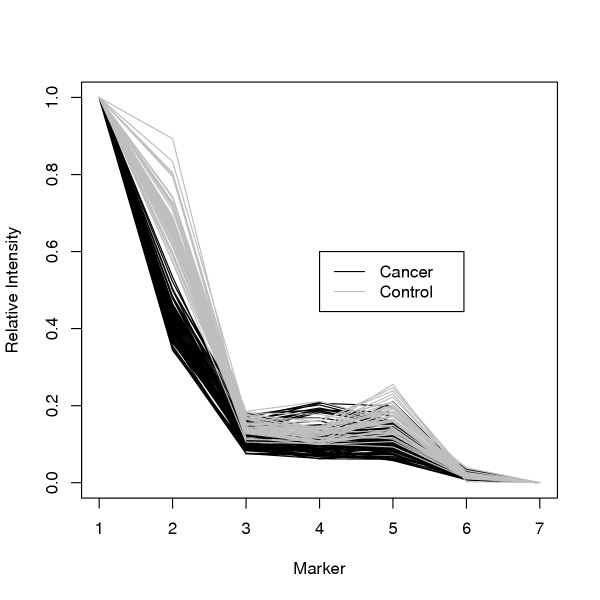
Test set classification of DS1 via 7 markers

The NCI-FDA website shows the chromosome consisting of *m*/*z *values {435.46, 465.57, 2760.67, 3497.55, 6631.70, 14051.98, 19643.41} was able to perfectly discriminate a test set drawn from the low resolution dataset, DSl. The 253 samples were randomly split into a training set of 81 cancer and 46 control individuals with the remainder forming a test set. Here we illustrate how the test set looks for these seven markers. Figure 1 shows the ratio of the second marker (molecular weight of 465.6) to the first (weight of 435.5) does an excellent job in separating the two types of samples in the test dataset. In the training set only two clusters were determined – one composed completely of cancer cases and the other solely of controls. In the test dataset one sample is misclassified (essentially because of its values on the remaining 5 markers) though it should be again pointed out that the number of misclassifications does vary by the order in which samples are processed and how the cases are split into test and training sets. Ten consecutive trials in which different test/training splits and ordering decisions were randomly made produced 1, 0, 0, 0, 0, 0, 0, 0, 5, and 1 errors (all misclassifications of normal as cancer) for this 7 marker model.

This exercise verifies that the model does quite well though it establishes that results do change with ordering and test/training set splits. Also, it confirms that results of 100% accuracy should be understood to depend upon the particular split and ordering. This may be of great importance when the goal is to develop tests with sensitivity and specificity exceeding 99% [12].

Next, the GA developed for this paper was then applied to these data with the expectation it should produce something like the 7 marker chromosome given above. After 7 generations, a chromosome was found that perfectly split the training set, but 7 clusters were required and 10 markers were used.

**Figure 2:**
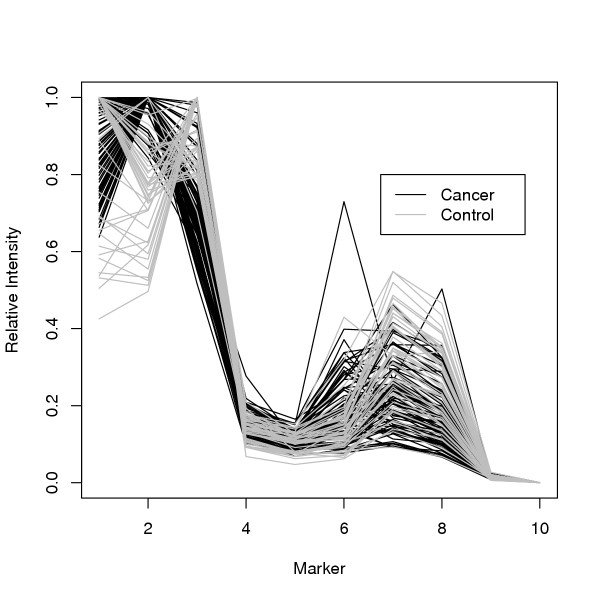
Test set classification of DS1 via 10 markers

The graph of test set classification, Figure 2, shows a less compelling picture of discrimination; the third marker is perhaps best (based on t-test p-values) at distinguishing samples. This marker corresponds to a molecular weight of 831.1 Daltons. To examine robustness the algorithm was run 9 more instances using different initial sets of 1500 chromosomes to try to get a sense of the variation in the algorithm's chosen models. The same ordering, test samples, and training samples were maintained.

In each case a perfectly discriminating (i.e. training set error of 0%) chromosome was found within a few generations. Table 1 shows that there is considerable variation in the test set accuracies given they were all produced by the same data. Further, the best discriminating single marker within the chosen chromosome shows little consistency. Also, the set of 10 generally shows a large number of clusters and markers – nothing very similar to the published model that contained only two clusters.

The results in Table 1 suggest there are many markers that are different in these data and it is easy to find classifiers that performs well – at least in the training set. However, it is also clear this creates a kind of algorithmic instability in that considerable variation in results can arise from the same training set data. Even if one uses a fixed training/test set division there is now a question of how does one decide which results to use? One could run the algorithm just once, but run the risk of choosing a not very good model (e.g. the model with 24 clusters and 16 markers). However, if the algorithm is run many times in the search for a good model, the reported sensitivity and specificity in the test set are likely to be biased. This question will be pursued further in the Discussion section below.

Table 1 and the preceding discussion of variation suggest this implementation of the algorithm might be improved by changing the procedure to favor models with fewer clusters/markers. In this way, those models that may overfit the training data are penalized and the number of perfect discriminators of the training set consequently reduced. A simple way of doing this is to alter the fitness function to penalize large numbers of clusters and/or markers. In the analysis above the fitness function was given as

Fitness = Accuracy

= % Correctly classified cases.

This could now be modified to

Fitness = % Correctly classified cases

- *p*_1_·# of clusters

- *p*_2_·# of markers

where *p*_1 _and *p*_2 _are non-negative penalization weights.

A resampling method was used to determine the performance of different parameter combinations of *p*_1 _among {0, .002, .005, .008} and *p*_2 _in {0, .001, .002}. Specifically, random training samples (chosen without replacement) were selected from the entire set of samples so the original training sample size of 127 was maintained with 81 cancer and 46 control spectra. Then, for a given pair of p_1 _and *p*_2 _values the GA was trained on this pseudo-random training set and a model chosen. The remaining cases that were omitted from the training set (81 cancer and 45 control) were then treated as a test set. We repeat this procedure for 50 randomly chosen training sets and examine the distribution of the test set classification accuracy for the different parameter combinations. The same 50 training and test sets were used for each set of *p*_1 _and *p*_2 _combinations. In addition to illustrating the test set accuracy, the number of clusters, the number of markers associated with the different GA models, and the proportion of times (out of 50) a perfectly discriminating chromosome was found (for the training set) are also indicated.

This procedure gives a sense for the performance of the algorithm for different parameter combinations. Another question concerns performance if model selection were incorporated into the procedure. This was assessed in the following way. For each of the 50 training sets, an additional 5-fold cross-validation determined which of the 12 *p*_1 _and *p*_2 _combinations performed best in terms of predicting the omitted cases (in the event of ties the model associated with the most restrictive *p*_1 _and *p*_2 _was chosen with *p*_1 _the first tie-breaker). The chosen parameters were then used with the entire training set to develop a model that was evaluated on the associated test set. As before, this procedure was performed on the same 50 training and test sets. In the tables that follow, the results for this model are labeled as "Best GA". These results are perhaps most representative of overall performance for the algorithm developed here.

As a means of comparison two other classification schemes were applied to the same bootstrap samples. Boosting is a general method of combining a weighted set of classifiers that each "vote" on the class of a sample in question with majority vote dictating the set's aggregate classification. It has been successfully used in classification of mass spectrometry data [4,13]. Here, the base classifier is a simple threshold classifier, e.g. if intensity at mass 245.8 ≤ 47.5 then classify as cancer, otherwise classify as normal. The general process by which the set of base classifiers is chosen is discussed at length in [4] and [14]. Here 150 was chosen as the number of base classifiers for the aggregate classifier and the algorithm generally followed that outlined in section 10.1 of [14]. The second algorithm used was PAM (Predictive Analysis for Microarrays), a shrunken centroid method of classification [15]. This method has been used for high dimensional microarray studies and is relatively easy to implement. Both methods require little operator assisted tuning to obtain a small feature set – an important consideration when conducting so many resamplings. For these methods the data were normalized (test and training sets normalized separately for each resampling) so each spectra had the same average intensity. Also, attention was restricted to those *m*/*z *values showing Bonferroni-corrected differential expression (calculated anew for each resampling). Computer code and information regarding the parameters and details of these methods are available on a webpage [16] with supporting documentation.

Table 2 shows the 25^*th *^and 75^*th *^percentiles for test set accuracy among 50 samples as described above. The two penalty parameters have the desired effect in reducing the number of clusters/markers but there is relatively small variation over the different parameter combinations. The GA models apparently perform a bit better than PAM and a little worse than the boosting method though all models have high accuracy. Some reviewers of these data [5,17], have questioned why the groups are so easy to classify and whether the entire *m*/*z *range should be used. The criticism centers around the strong signals that are present in very low *m*/*z *values (e.g. 2.79 and 245.54 Daltons) that are speculated to be products of experimental procedures rather than reflective of biological differences. Other investigators [2] routinely exclude the lower end of the spectrum (less than 1500 or 2000 Daltons) as they feel it too contaminated by matrix and other effects to be clearly interpretable. As a result of these concerns the experiment was rerun with the *m*/*z *range restricted to be greater than 1500 Daltons.

Table 3 is based upon the *m*/*z *restricted dataset and shows evidence of greater spread among the different GA models – those with *p*_1 _= 0 or .008 do not appear to do as well as *p*_1 _= .002 or .005. With no penalty on the number of clusters one sees very high dimensional models (median number of clusters > 90), perfect training set performance every time, and relatively poor test set performance indicating some type of penalization is necessary. Increasing the value of *p*_2 _has the desired effect of yielding more parsimonious models without an obvious decline in performance. As before, the GA models seem to perform better than PAM but less well relative to the boosting model.

Next, results from the high resolution dataset are presented. The data require preprocessing. Some samples contain raw data from approximately 370,000 *m*/*z *values in the 700 – 12,000 Dalton range while other samples have about 330,000 data points. This discrepancy is particularly worrisome as the cancer samples appear more likely to have fewer datapoints. The information presented at the NCI-FDA website and in [12] includes some discussion of how the data were aggregated. The implementation in this work is similar to that described at the NCI-FDA website – details are available at a webpage containing supporting material [16]. After aggregation, the resulting spectra containing 7106 points were normalized to have the same average intensity. We note (data not presented) that while the models for the high resolution dataset on the NCI-FDA website have relatively good test set performance (accuracy of about 95%) they entail a large number of clusters – typically between 30 and 50. This is in contrast to the model reported for the low resolution DS1 Ciphergen data that had two clusters.

The results in Table 4 are quite similar to those reported for DS1 in the *m*/*z >*1500 range in that poorly performing high dimensional models are associated with *p*_1 _= 0 and the GA appears to again perform at an intermediate level.

## Discussion

As implemented here, the genetic algorithm without penalties produces a large number of chromosomes that can perfectly discriminate a training set of the type considered here. For the last two analyses (DS1 with *m*/*z *> 1500 and DS2) those models produced with *p*_1 _= 0 are associated with a large number of clusters (median ≥ 90), indicating that many clusters have only 1 or 2 individuals. As demonstrated above, a resampling approach shows that models with large numbers of clusters will generally not perform as well as more parsimonious chromosomes and the use of penalization parameters greatly improves performance.

While this modification results in better models it does not address the other fundamental question of how to choose a final model. Because of their reliance on random choices GA models can and, for these data, will present very different solutions from a given dataset. Therefore, even if one uses a supplemental cross-validation process to choose some ideal set of penalization parameters many different models can be chosen by running the GA repeatedly. This is in contrast to many other means (e.g. boosting, discrimination methods) that have no such reliance on random processes and will produce the same answer given a fixed dataset and parameters. Some methods such as decision trees and PAM employ cross-validation as part of their fitting process and therefore do have a random component, but the results are not nearly so variable, at least for the data considered here. Next we explore some consequences when the GA is repeatedly applied to a fixed training and test set with the goal of finding "best" or superior models. Such repeated examination of test set performance violates the principal of evaluating the test set only after the model has been selected [14].

### Consequences of repeated model fitting

Given a model developed on a training set, the performance of such a model on an independent test set is an unbiased estimate of its performance when exposed to a subsequent group of unlabeled cases that are generated by the same process. However, the situation becomes more complicated when a collection of models is considered. It is generally not true that the best performing model (judged by which model attains highest test set accuracy) will reproduce similar results on a yet another group of cases. Essentially, while every model has a true error rate, its performance on a particular test set is a function of both the true error and random variation. The best performing model is likely the beneficiary of positive random variation that is unlikely to be repeated in application to yet another set of data. In this sense the best performing model has an underestimated error rate when the selection of the best model is performed via repeated examination of a test set. We present a final set of bootstrap based analyses to illustrate the degree of bias.

For DS1, 50 runs of the following type of experiment were performed. First, a bootstrap sample of size 253 with 162 cancer and 91 controls is drawn (the cancer and control individuals were bootstrapped separately from their respective cohorts). This is denoted as *X*^*b *^while the original cohort is *X*. This bootstrap sample is then split into training (81 cancer, 46 control) and test sets (81 cancer, 45 control). On the training part of the bootstrap sample the GA is run with *p*_1 _= .005 and *p*_2 _= .001. These parameters were chosen as they seemed to perform relatively well in Tables 2, 3, and 4 and they generally employed a smaller number of clusters and markers. The GA produced a model associated with this particular bootstrap sample, denoted 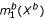. The performance of that model was then evaluated by the error rate in the test set portion of the bootstrap sample, denoted 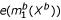. Because the GA process produces different estimates when run on the same data due to randomness as described above, the model-fitting process is then repeated 19 more times on the same bootstrap sample to obtain 20 different models 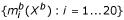 and 20 different measures of performance 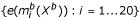. The order of the training set and random sampling decisions made by the GA were allowed to vary though the composition of the test and training set were fixed for a given bootstrap sample. The best model, denoted 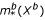, was chosen as that among the 20 with lowest classification error, 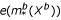. In the event of a tie, the number of clusters served as a tie-breaker (smaller is better). This procedure is meant to mimic the idea of applying 20 models to the test set and settling upon the best one. To get an idea of the bias in estimation error we then examine how the chosen, best model performs for the original cohort of 253 cases – this error rate is denoted as 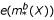 and the bias estimated by 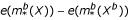. This procedure was performed 50 times and one obtains an estimate of the distribution of the bias from


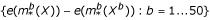


in 1) DS1 using the whole *m*/*z *range, 2) DS1 restricting the range to *m*/*z *> 1500, and 3) DS2 with 700 <*m*/*z *< 12000 (using different corresponding sample sizes). The use of the bootstrap to assess bias in this way conforms to the notion of treating the full sample distribution like a population distribution and the bootstrap sample distribution like the full sample distribution – see chapter 10 of [18].

The results in Table 5 show the degree of bias is relatively modest in the first dataset – on the order of 2% and somewhat higher (median of 4–5%) in the other data under consideration. This may not be of great practical import unless one is particularly concerned that the specificity be near 100% to justify using such tests on the basis of widespread diagnostic testing [12]. The degree of bias is influenced by, among other things, the number of times the test set is interrogated – here the figure used was 20 and it may be that greater bias is associated with increased searching. This analysis could also have been performed by splitting the data into 3 datasets (training, test, and bias assessment groups) though these datasets are small enough that the bootstrap approach was preferred in that it makes more efficient use of the data.

### Generalizing results

There is considerable controversy regarding these ovarian cancer datasets – particularly with respect to whether the multitude of models with high or perfect sensitivity and specificity are more the result of rich complexity reflecting true biological variation [19,20] or flaws in experimental design [5,17]. While it is of paramount importance to know if true biological difference or flaws in experimental design are primarily responsible for the ease with which classification algorithms can separate the cancer and normal spectra (especially the low resolution dataset with 0 <*m*/*z <*20000 Daltons), the algorithms' performances will not change regardless of the answer to this question. Therefore, in the limited context of algorithmic performance considered here this critical issue is of secondary importance and not addressed. This observation indicates that these algorithmic analyses may still be valuable even if one believes the datasets to be flawed.

It is interesting to note how the GA performed on these three different datasets and speculate on its performance in other circumstances. The results in Table 5 regarding bias arising from multiple applications of the GA do vary somewhat among the three datasets under consideration. Because the spectra in the full, low resolution dataset (0 <*m*/*z *< 20000 Daltons) are easiest to correctly classify (as seen in Table 2) this dataset shows the smallest degree of bias arising from multiple applications of the GA – the median bias is about 2% in Table 5. Essentially the bias is low because the normal and cancer samples are so distinct and many models do very well. This is the case even though the multiple models may look very different from one another and use different primary *m*/*z *values to discriminate; in this case the bias is low because virtually all the dissimilar models do quite well.

The truncated, low resolution dataset (*m*/*z *> 1500 Daltons) was used to exclude the lower mass values that some [21] believe are difficult to interpret. Exclusion of these masses made the spectra harder to classify (see Table 3) and the associated bias in Table 5 was greater. The GA's performance in the high resolution dataset showed perhaps an intermediate level of difficulty in correctly classifying spectra (Table 4) and a corresponding intermediate degree of bias. The results suggest that as the spectra become easier to classify, the degree of bias due to repeated model fitting declines. This generalization is speculative in that it is based solely on these three related datasets and should be investigated in other datasets. Also, it should be pointed out that bias may be quite low in situations where there are only a few *m*/*z *values that can distinguish spectra. In this case one could speculate that repeated model fittings may identify primarily the same chromosome and therefore lead to very little bias.

## Conclusions

This paper presents a genetic algorithm based on descriptions in earlier work. It was difficult to exactly reproduce performance of the original algorithm because important aspects were not well described and questions directed to the associated website were unacknowledged. Consequently, the GA's implementation described here is likely different than that made to produce the published findings. In particular, there are ambiguities concerning the manner in which more "fit" chromosomes are chosen to produce the subsequent generation, how chromosomes of different lengths may be produced and combined, and the possible use of penalization or other means to obtain parsimonious models. Despite these potential differences some aspects of the original algorithm's performance are likely shared with those of the model developed here.

Some modification of the algorithm to guard against overfitting seems necessary to obtain good performance. In particular, defining the fitness function simply as training set classification accuracy produces models with too many clusters. Here, a penalization based on the number of clusters and markers was imposed that improved algorithmic performance. A cross-validation procedure was incorporated to choose the penalization parameters – this resulted in algorithmic performance similar to other classification schemes. Results based on this type of algorithm should be accompanied by a clear description of how individual models are generated, e.g. what penalization parameters or other means of reducing the number of clusters are included and how they were chosen.

There is randomness and lack of reproducibility in model performance that depends on order of cases, random choice of initial chromosomes, and how the fitness function determines the subsequent chromosomes. Consequently, for a fixed training dataset, the algorithm can produce many chromosomes that perform well simply by repeatedly running the algorithm. There may be a temptation to use the algorithm repeatedly and evaluate test set performance to select the final model(s). While this can be a problem for classification algorithms in general, the random characteristics of this procedure may make it especially hard to resist. Here we saw some sense of the bias resulting from, such an approach. As the discussion regarding bias demonstrates, the reported sensitivities and specificities cannot be adequately assessed without very detailed description of the models' discovery. In this sense, those who employ such a scheme must supply complete information regarding the entire process used to choose the given models and users of algorithms that have this property of producing multiple models from a fixed dataset must be aware of this potential bias.

Overall, once modifications have been incorporated to address the overfitting concerns, the algorithm's performance seems comparable to other methods. It should be noted that the final models produced by this GA are of a simple to interpret form that may be based on a small number of markers and clusters. This simplicity is not necessarily present for other algorithms (e.g. boosting, neural nets, support vector machines). This algorithm seems a reasonable option for creating discrimination models though it does have disadvantages that might guide analysts to choose a different approach.

## Methods

### Data sources

The low resolution dataset, DS1, was obtained from a ProteinChip Biomarker System-II (PBS-II) surface-enhanced laser desorption ionization time-of-flight (SELDI-TOF) instrument produced by Ciphergen Biosystems, Inc. of Fremont, CA, USA using a WCX2 ProteinChip array, also produced by Ciphergen. Further details regarding sample handling and preparation are not readily available from the NCI-FDA website. While data from earlier low resolution datasets were available from the NCI-FDA website, these data (labeled 8-7-02) were chosen because the baseline does not appear to have been subtracted. As discussed by others [17] it does not appear possible to reproduce the original results of the genetic algorithm after baseline subtraction has been performed.

The high resolution dataset, DS2, was obtained from a hybrid quadrupole time-of-flight mass spectrometer (QSTAR pulsar *I*, Applied Biosystems, Inc. Framingham, MA, USA) modified to read the WCX2 ProteinChip. Additional information regarding handling and preparation of samples is available in [12].

Normalization of the two datasets was performed differently. For the low resolution data the spectra were rescaled linearly so the smallest value was 0 and the largest was 1. This was described in an earlier document on the NCI-FDA website (since removed) and was the approach described in [17]. This transformation has no effect on the genetic algorithm since additional rescaling is done within each individual spectrum on a chromosome by chromosome basis. This may have some effect (relative to performing no normalization) on the boosting and PAM algorithms but it is likely to be quite small as the maximum value for each spectrum was 100 (except one which reported a max value of 99.75) and the minima lay between 3.75 and 3.95 – so the effect was nearly one of applying the same transformation to each spectrum. The PAM and boosting algorithms were implemented after an additional normalization step that equalized the average intensity for each spectrum.

For the DS2 data, once the raw values were aggregated into 7106 bins the spectra were normalized to have the same average intensity. Here it seemed necessary to try to address the fact that the intensities for samples processed later were generally less than those processed earlier – see the QC document on the NCI-FDA website and [12]. Again, the normalization has no effect for the genetic algorithm. For the other algorithms it seemed important to try to address this temporal effect.

### Data processing

Computing for all the classification algorithms (GA, boosting, and PAM) was done using the R programming language. Results for the PAM algorithm were obtained using the pamr package available from the R website [22]. On the website housing supporting information for this paper [16], full details are available showing the code and steps necessary to reproduce the findings presented in this paper.

## Figures and Tables

**Table 1:** Variation in results of GA applied 10 times

Generations	Test Set Errors	Clusters	Markers	Primary *m*/*z*
7	6(5%)	7	10	831.1
14	10(8%)	24	16	617.7
9	2(2%)	8	19	246.7
6	4(3%)	21	7	632.6
13	8(6%)	13	14	226.9
5	9(7%)	7	20	42.6
9	4(3%)	5	11	831.1
9	7(6%)	30	10	617.7
9	1(1%)	7	11	786.5
7	1(1%)	5	17	42.6

**Table 2:** Accuracy percentiles and characteristics of models from 50 cross-validation samples of DS1, *m*/*z *> 0

Algorithm	Test Set Accuracy 25^*th*^, 75^*th *^Percentile	Median # Clusters	Median # Markers	Proportion of Perfect Chromosomes
GA(*P*_1 _= 0, *p*_2 _= 0)	.96, .98	12	15.5	1.0
GA(0, .001)	.95, .99	12.5	11	1.0
GA(0, .002)	.95, .98	12.5	10	1.0
GA(.002, 0)	.96, .98	7	16	.98
GA(.002, .001)	.97, .98	7	12.5	1.0
GA(.002, .002)	.96, .99	8.5	9	.98
GA(.005, 0)	.96, .98	6	15	.92
GA(.005, .001)	.96, .98	5	11	.92
GA(.005, .002)	.96, .99	6	8	.94
GA(.008, 0)	.96, .98	3	15	.72
GA(.008, .001)	.97, .99	4	9	.88
GA(.008, .002)	.97, .99	4	8	.84
Best GA	.97, .99	6	12	.94
Boosting	.99, 1.0	NA	NA	NA
PAM	.93, .97	NA	NA	NA

**Table 3:** Accuracy percentiles and characteristics of models from 50 cross-validation samples of DS1, *m*/*z *> 1500

Algorithm	Test Set Accuracy 25^*th*^, 75^*th *^Percentile	Median # Clusters	Median # Markers	Proportion of Perfect Chromosomes
GA(*p*_1 _= 0, *p*_2 _= 0)	.83, .87	90	11	1.0
GA(0, .001)	.80, .87	93	11	1.0
GA(0, .002)	.82, .87	92.5	9	1.0
GA(.002, 0)	.87, .92	12	19	.26
GA(.002, .001)	.90, .93	14	13	.38
GA(.002, .002)	.88, .92	13	7.5	.20
GA(.005, 0)	.87, .92	5	20	.02
GA(.005, .001)	.87, .90	5	10	.02
GA(.005, .002)	.87, .92	5	6.5	0
GA(.008, 0)	.85, .90	3.5	18.5	0
GA(.008, .001)	.85, .89	4	8.5	0
GA(.008, .002)	.85, .90	4	6	0
Best GA	.87, .91	7	10	.12
Boosting	.93, .97	NA	NA	NA
PAM	.80, .84	NA	NA	NA

**Table 4:** Accuracy percentiles and characteristics of models from 50 cross-validation samples of DS2, 700 <*m*/*z *< 12000

Algorithm	Test Set Accuracy 25^*th*^, 75^*th*^	Median # Clusters	Median # Markers	Proportion of Perfect Chromosomes
GA(*p*_1 _= 0, *p*_2 _= 0)	.70, .80	91.5	17	1.0
GA(0, .001)	.63, .75	100	11	1.0
GA(0, .002)	.61, .74	100	10	1.0
GA(.002, 0)	.89, .93	22.5	19.5	.86
GA(.002, .001)	.88, .93	22	14.5	.90
GA(.002, .002)	.87, .91	22	8	.86
GA(.005, 0)	.88, .93	7	19	.20
GA(.005, .001)	.88, .93	7	11	.20
GA(.005, .002)	.87, .93	7	6	.16
GA(.008, 0)	.86, .91	5	18	.02
GA(.008, .001)	.86, .91	5	10	.04
GA(.008, .002)	.86, .91	5	4	.02
Best GA	.88, .93	7	12	.24
Boosting	.92, .95	NA	NA	NA
PAM	.83, .86	NA	NA	NA

**Table 5:** Percentiles relating to bias of repeated examinations of a test set

	Percentile				
	
	10^*th*^	25^*th*^	50^*th*^	75^*th*^	90^*th*^
DS1, *m*/*z *> 0					
Bootstrap smallest error: 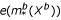	0.0	0.0	0.0	0.0	0.0
Original cohort error: 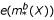	.01	.01	.02	.03	.05
Estimated bias: 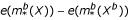	.003	.012	.020,	.032	.051
DS1, *m*/*z *> 1500					
Bootstrap smallest error: 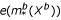	.02	.02	.03	.05	.06
Original cohort error: 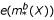	.07	.07	.08	.11	.13
Estimated bias: 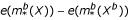	.02	.04	.05,	.07	.10
DS2, 700 <*m*/*z *< 12000					
Bootstrap smallest error: 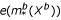	.01	.02	.03	.04	.05
Original cohort error: 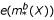	.04	.05	.06	.08	.10
Estimated bias: 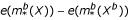	.01	.03	.04,	.06	.07
